# Analysis of queries sent to PubMed at the point of care: Observation of search behaviour in a medical teaching hospital

**DOI:** 10.1186/1472-6947-8-42

**Published:** 2008-09-24

**Authors:** Arjen Hoogendam, Anton FH Stalenhoef, Pieter F de Vries Robbé, A John PM Overbeke

**Affiliations:** 1 Department of Medicine, Division of General Internal Medicine, Radboud University Nijmegen Medical Centre, Nijmegen, the Netherlands; 2 Department of Medical Informatics, Radboud University Nijmegen Medical Centre, P.O. Box 9101, 6500 HB, Geert Grooteplein 21, Nijmegen, the Netherlands

## Abstract

**Background:**

The use of PubMed to answer daily medical care questions is limited because it is challenging to retrieve a small set of relevant articles and time is restricted. Knowing what aspects of queries are likely to retrieve relevant articles can increase the effectiveness of PubMed searches. The objectives of our study were to identify queries that are likely to retrieve relevant articles by relating PubMed search techniques and tools to the number of articles retrieved and the selection of articles for further reading.

**Methods:**

This was a prospective observational study of queries regarding patient-related problems sent to PubMed by residents and internists in internal medicine working in an Academic Medical Centre. We analyzed queries, search results, query tools (Mesh, Limits, wildcards, operators), selection of abstract and full-text for further reading, using a portal that mimics PubMed.

**Results:**

PubMed was used to solve 1121 patient-related problems, resulting in 3205 distinct queries. Abstracts were viewed in 999 (31%) of these queries, and in 126 (39%) of 321 queries using query tools. The average term count per query was 2.5. Abstracts were selected in more than 40% of queries using four or five terms, increasing to 63% if the use of four or five terms yielded 2–161 articles.

**Conclusion:**

Queries sent to PubMed by physicians at our hospital during daily medical care contain fewer than three terms. Queries using four to five terms, retrieving less than 161 article titles, are most likely to result in abstract viewing. PubMed search tools are used infrequently by our population and are less effective than the use of four or five terms. Methods to facilitate the formulation of precise queries, using more relevant terms, should be the focus of education and research.

## Background

Searching medical information on the internet has rapidly gained a place in daily medical care. Many sources are available for answering patient-centred questions. One of the main sources for medical information is Medline with PubMed as search engine. A major limitation of PubMed is that it takes 30 minutes on average to find information and appraise the literature critically [1]. When searching for patient-related problems at the point of care the physician wants to find information quickly [2,3]. Critical appraisal is the time-consuming step in the process. It is difficult to reduce the time needed to appraise the literature, which depends on the experience of the reader. However, reducing the number of articles that have to be appraised can reduce the search time significantly. It is difficult to retrieve only relevant articles from the large PubMed database as PubMed searches are characterised by retrieval of a vast number of article titles in very broad searches and a limited number of article titles in narrow searches [4]. The simplest method for reducing the numbers to read is to increase the number of terms in a query. Other PubMed tools available to the searching physician that can limit the number of retrieved articles are Boolean operators, Mesh and limits. A special set of tools advocated by evidence-based medicine handbooks [5,6], Clinical Queries, were designed to help in finding answers to clinical questions [7-12]. Many combinations of tools and term counts are possible and the results are often difficult to predict. As PubMed does not sort articles by relevance, the number of articles retrieved by a query plays a crucial role. Evaluation of hundreds of articles is useless when time is critical, but there is no information about the number of articles that can be scanned at the point of care. It is possible to issue several queries, increasing the accuracy of the query step by step, but this process is too time-consuming for use during daily medical care. The physician should be able to find a potentially useful article within one or two queries, leaving enough time for critical appraisal. Observation of the search process during daily medical care is crucial for identifying the tools that actually work in this setting. We therefore created an online information portal that could monitor the complete search process without interfering with the search. Physicians working at our teaching hospital are accustomed to using online information sources and they have all received some education in evidence-based medicine. They are therefore likely to use a wide array of queries and search tools. We performed an observational study of queries sent to PubMed during daily medical care to answer the following questions. To what extent are search tools used, and does the use of these tools improve article selection for further reading? How many articles should be retrieved by a query to enhance the chance that one will be selected for further reading? What is the relationship between the number of terms, the articles retrieved by a query and abstract selection? We use abstract and full-text selection as parameters for success of a query.

## Methods

### Population and measuring tool

As part of an ongoing study of sources used for retrieving medical information we developed a web portal. This portal gives access to PubMed, two online medical textbooks (UpToDate, Harrison's Online) and a Dutch pharmacotherapy database. All residents and specialists in internal medicine selecting PubMed or UpToDate from our hospital information system were automatically linked to our portal.

### PubMed interface

**Figure 1:**
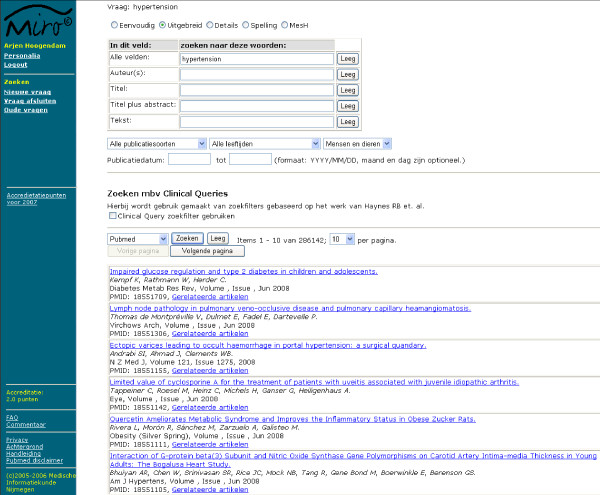
**PubMed search interface**. The advanced search options are available in the upper section. Besides search field descriptions (title, abstract and text word), several filters are available: publication types, age criteria, humans/animal and Clinical Queries filters. The PubMed search result for hypertension is shown in the lower section.

To enable all aspects of the use of PubMed to be registered we built our own interface that accesses PubMed through e-utils [13]. E-utils gives access to full PubMed functionality – queries are handled exactly as they are in the original PubMed website – but it delivers the data in XML, which allows them to be recorded in a database. The XML data need to be translated into web pages to be readable by users. To mimic the functionality of PubMed, most of the special search options relevant to patient-related searches [5,6] were copied in our interface (Mesh database, details, a selection of limits (Publication date, publication types, human or animal and ages) and spelling (Figure 1). All queries were recorded along with the use of the different search options, the articles that were selected for abstract reading and the articles that were selected for full-text reading.

### Search process

Every search was started by entering a query and selecting an information source. The sending of the first query about a problem was marked as the start of the search. During the search, all queries were recorded, as well as the database that was consulted. After the search was completed, the users were asked to select the situation that had led to the search (direct patient contact, patient rounds, scientific research, review/study, preparing talks, not specified).

### Query characteristics and evaluation of search result

All queries sent to PubMed regarding patient-related problems (direct patient contact, patient rounds) were selected for analysis.

### Full-text and abstract selection as endpoints

Queries resulting in the selection of abstracts and/or full-text articles containing information that can be used to answer a question are considered as adequate queries that contribute to the search process. Ideally, the answer can be found in a single source by a single query, but in practice an answer to a question may be composed of multiple bits of information from several sources. Queries retrieving multiple articles that contain parts of the answer are therefore just as useful as queries that result in a single article containing the answer. The selection of an abstract containing information that contributes to the question is therefore a marker for the quality of the query. As the selection of abstracts is based on the title of articles some selected abstracts may not have attributed to the answer. This is a potential source of bias. Asking participants to rate the value of each selected abstract would result in interference with the search process. Participants would also refuse to use such an information source for an extended period. Interference with the search process is likely to result in bias, so the parameters of success of a query have to be extracted from search-related data. As it is unlikely that abstracts containing no information related to the question will be selected for full-text reading, selection of a full-text article is a marker for relevance of the abstract. However, not all abstracts contain links to full-text articles. Full text availability is therefore a possible confounder. Selection of irrelevant abstracts and online unavailability of full-text articles as sources of bias are unlikely to be related, as full-text availability does not depend on the relevance of the abstract to the question. If the results for full-text selection are comparable to those for abstract selection, both sources of bias are excluded. We therefore present data for both abstract and full-text selection.

### Relationship between number of terms and abstract selection

PubMed combines all terms with the Boolean operator "AND". The use of terms without operators is therefore equivalent to combining all terms with the "AND" operator. Using more terms will therefore lead to fewer articles in the article result list. Most searches on the internet use only the "AND" operator, if any Boolean operator is used at all[14]. The number of articles retrieved by such a natural language query is directly related to the number and relevance of the terms used. To determine the relationship between the number of terms used in a query, the number of articles retrieved by a query and abstract selection we selected all queries containing natural language with or without the use of the "AND" operator. Queries containing the "OR" or "NOT" operator or Mesh terms were excluded. Terms were identified as words separated by a space. The "AND" operator was not counted as a term. The use of more than six terms in a query was too infrequent to merit detailed analysis. Evaluation of the relationship between term count and query result was therefore limited to queries containing fewer than seven terms. Terms that reflect the clinical question and are likely to retrieve relevant information are regarded as relevant in our study. Abstracts and full-text articles that contain information contributing to the question are considered relevant to the question.

### Relationship between terms, articles retrieved and abstract selection

Only queries containing natural language that retrieved one or more articles were selected to demonstrate the relationship between term count and number of articles retrieved. Many terms will yield a small set of articles and a few terms will yield a large set. The number of articles retrieved by random terms therefore follows a logarithmic distribution. Combining several terms will not alter this distribution. As logarithmic numbers are difficult to interpret we divided the number of articles retrieved by a query into 14 equal intervals (average of 180 queries per category).

### Statistics

Frequencies were used to summarize data. Significance was determined by the Chi-Square statistic using SPSS, release 14.0.2.

### Ethical approval

No ethical approval was needed for this study, which involved no patients. All participants in our study consented to the use of search-related data for scientific research. Data were only collected if participants logged in at our internet portal.

## Results

### Query characteristics

The use of PubMed was monitored from October 2005 until January 2007. During this period 3205 distinct queries were sent to PubMed. These queries were related to a total of 1121 patient-centred questions posed by 94 specialists and residents in internal medicine. In 999 (31%) of the 3205 queries an abstract was selected for further reading (Table 1). In 456 (14%), full-text was selected for further reading. The "AND" operator was frequently used, but as PubMed links all words in the query with "AND", the use of this operator is not necessary. Other operators, wildcards, Mesh or limits where used in 321 (10%) of the 3205 queries. When these search tools were used, 126 (39%) of 321 queries resulted in the selection of abstracts for further reading.

**Table 1:** Aspects of queries sent to PubMed.

Aspects	All queries (N = 3205)
	n(%)

AND used *	1409(44)
OR used *	22(0.7)
NOT used *	6(0.2)
wildcard used †	65(2)
Mesh or Limits used ‡	252(8)
Query result positive §	2521(79)
Abstract selected ||	999(31)
Full text selected ||	456(14)

* Boolean operators. † Asterisk functions as wildcard. ‡ All limits or Mesh terms were identified by the use of square brackets in a query. § One or more article titles retrieved by query. || Queries that resulted in the selection of abstract or full-text articles for further reading.

### Evaluation of the search result

The query result is displayed as ten titles per page by default. To display more results, participants had to select the next page of results or change the number of articles displayed on screen. In 2625 (81.9%) of the 3205 queries only the first ten titles were viewed and no consecutive pages were selected (table 2). In 1959 (61.1%) of the queries, more than 10 articles were retrieved. Among these 1959 queries, only 20% of the retrieved articles were actually evaluated.

**Table 2:** Total number of titles that were displayed on screen by PubMed as a result of a query.

Titles*	Queries (N = 3205)
	n(%)†

10	2625(81.9)
20	284(8.9)
30	111(3.5)
40	62(1.9)
50	31(1.0)
>50	92(2.9)

*Total number of titles of articles in the query result list that were displayed on screen. If multiple pages of article titles were viewed for a single query the total number of pages presented on screen was calculated by adding the results displayed per screen. †Percentages do not add up to 100% because of rounding.

### Relationship between number of terms and abstract selection

**Figure 2:**
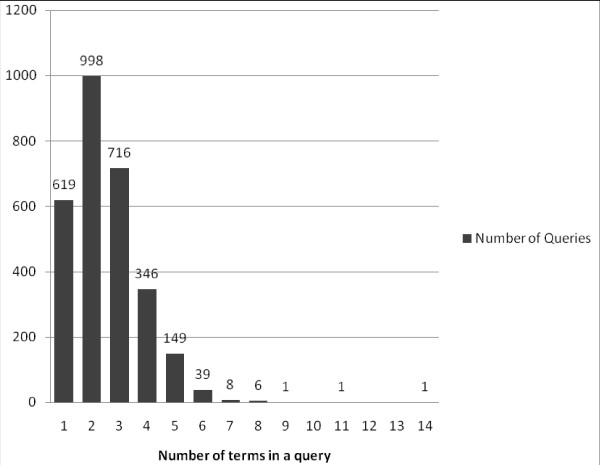
**Distribution of term count in PubMed queries**. Selection of 2884 queries containing no Mesh headings, limits, wildcards or special operators, "AND" operator allowed.

After selecting queries containing no Mesh, limits, wildcards or special operators ("AND" operator allowed), 2884 natural language queries remained. On average, 2.5 terms excluding operators were used in these queries. In 1617 (56%) of the 2884 queries only 1 or 2 terms were used, and 2828 (98%) consisted of fewer than 6 terms (Figure 2). The relationship between the number of terms used and the proportion of queries leading to the selection of abstracts is shown in Table 3. Using more terms increases the risk of finding no articles at all. The percentage of queries yielding no articles slowly rises to 33% as the number of terms in a query rises to 6. Increasing the number of terms in a query increases the proportion of queries leading to the selection of abstracts from 13% (one term) to 43% (five terms). The proportion of queries leading to the selection of articles for full-text reading reaches a plateau of 23% when more than four terms are used.

**Table 3:** Queries that yielded no articles in the PubMed result list, queries that resulted in abstract selection and queries that resulted in full-text selection in relation to the number of terms used.

Terms	Query result		
	
	No articles retrieved by query	Abstract selected	Full-text selected
	n/N(%)	n/N(%)	n/N(%)

1	101/619 (16)	79/619 (13)	28/619 (5)
2	197/998 (20)	291/998 (29)	108/998 (11)
3	174/716 (24)	277/716 (39)	131/716 (18)
4	86/346 (25)	145/346 (42)	80/346 (23)
5	42/149 (28)	64/149 (43)	32/149 (21)
6	13/39 (33)	12/39 (31)	9/39 (23)

Selection of 2867 queries containing no Mesh headings, limits, wildcards or special operators, "AND" operator allowed. Queries containing more than 6 terms excluded.

### Relationship between terms, articles retrieved and abstract selection

**Figure 3:**
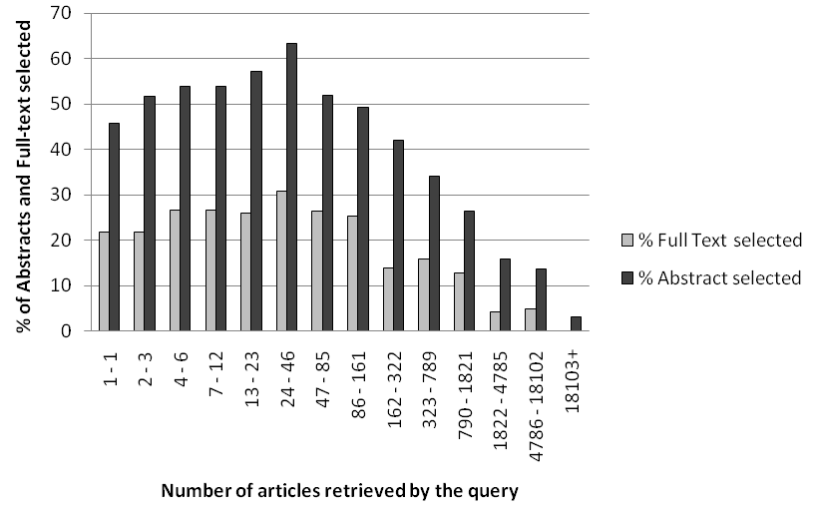
**Percentage of queries leading to abstract or full-text reading in relation the number of articles retrieved by a query**. Selection of 2521 queries that yielded one or more articles.

The percentage of queries resulting in abstract or full-text viewing as a function of the number of articles retrieved by a query is shown for 2521 queries that yielded one or more articles. The percentage of queries that led to abstract selection remains above 49% when 2–161 articles are retrieved (Figure 3) and rapidly declines thereafter. The relationship between term count and abstract selection could be entirely attributable to the number of articles retrieved by a query. To determine the magnitude and dependence of each of these two parameters we looked at abstract selection in optimal queries for term count and/or number of retrieved articles (table 4). These results show that retrieving 2–161 articles is a better predictor of abstract-viewing than using four to five terms in a query, but the two factors have independent effects as most queries lead to abstract selection if both conditions are met.

**Table 4:** Relationship between optimal term count and optimal number of articles retrieved by a query, cross-tabulated by abstract selection.

	4 or 5 Terms	Fewer or more than 4 or 5 Terms	
	AS/NQ(%)†	AS/NQ(%)†	NQ†

2–161 articles retrieved by query	161/254(63)	411/807(51)	1061
1 or more than 161 articles retrieved	48/113(42)	248/1080(23)	1193
Total NQ	367	1887	2254

Selection of 2254 queries resulting in one or more retrieved articles and containing no Mesh headings, limits, wildcards or special operators, "AND" operator allowed. †AS = Queries leading to Abstract Selection. NQ = Total Number of Queries in category.

## Discussion

Physicians at our university hospital, searching for patient-centred problems in PubMed, do not differ much from the general public using search engines such as Google[14]. They make very simple queries, containing two to three terms on average. In consequence, many queries yield a list of more than 161 articles, which are not further evaluated for relevance. The use of PubMed search tools was very limited and the performance of these tools was comparable to the use of more than three terms in a query.

### Query characteristics

Our participants used two to three terms on average. Previous research found three terms [15]; this difference may be because we did not count Boolean operators as terms, unlike the authors of [15]. As all searches are connected to patient-related problems we expected the queries to contain more terms to describe the question more adequately. Another reason for expecting more terms in a query is that general questions are relatively easy to find in information sources containing aggregated data, such as evidence-based textbooks. Physicians are therefore advised to use reviews and studies as consecutive last steps in the search process when other sources cannot provide an answer [16]. This makes it unlikely that the questions that were looked up in PubMed were general in nature. The more likely reason for lack of detail is that despite all recommendations for constructing proper queries in evidence-based medicine [5,6], physicians do not take the time to construct such queries. A study by Ely et al showed that physicians could not answer 41% of pursued questions. Analysis of unanswered questions showed that it was possible to answer a proportion of unanswered questions if queries were reformulated, better describing the question[17,18]. It has been shown that training courses in evidence-based practice improve search skills considerably [19,20]. Our results show that term count and number of retrieved articles in the query result have independent effects. If using more terms only reduced the number of irrelevant articles, then term count should not have an independent effect. Using more terms related to a question must therefore also increase the number of relevant articles. This is most likely to be related to a more precise description of the question. Although the percentage of queries yielding no articles rises slowly with the use of more terms, it does not have a negative effect on abstract selection up to at least 6 terms. Physicians should therefore be urged to use enough terms, describing the question accurately, and should not fear that this will yield too few articles. As our population is familiar with evidence-based searching, the question is why they do not use advanced search methods. One possible reason is that search tools are not on the main page of our portal and PubMed but require navigation to special search sections. As truly effective tools are likely to be used even when they are difficult to locate, this may not be a valid argument. Another reason might be that participants do not use the PubMed search tools effectively. Our participants selected fewer abstracts with search tools than with the use of four or five terms, and this might be related to improper use of the search tools. Tools that are effective in laboratory situations but are difficult to use properly during daily medical practice are inefficient for this type of search and should not be advocated for use initially. A final reason might be that other search engines do not require the use of advanced search methods and physicians try to search in the way most familiar to them. Examples of such search engines, delivering ranked results, are Google, Google Scholar and Relemed [21]. Because these search engines perform relevance ranking they can be used effectively with natural language queries. The relative ease of Google searching has led to a publication advocating the use of Google to help solve patient-related diagnostic problems [22]. The question is whether physicians should be taught to use these search engines or to use better search techniques in PubMed. One argument against Google is that there are several fundamental issues regarding the reliability of the information retrieved and the validity of the ranking method [23]. More importantly, formulating accurate clinical questions and translating them into well formed queries, with or without the use of additional search tools, is likely to increase the accuracy of the search result regardless of the search engine used.

### PICO as a method to improve a query

One method for translating clinical questions into accurate queries is the PICO method. This method can help to build adequate queries regarding patient-related problems [5,6,24,25]. In the PICO method the physician is instructed to describe the patient-related problem in three to four concepts (Patient characteristics, Intervention, Comparison and Outcome). This technique was designed for questions regarding therapy but can be adapted to questions about diagnosis. Using the PICO formulation is likely to result in better queries, limiting the number of results. Although the majority of questions posed by clinicians are related to treatment and diagnosis that can be translated into PICO, many clinical questions cannot be translated into PICO. For example, questions regarding prognosis, the etiology of a disease, economic consequences, biochemical compounds, physiological principles, pathology, genetics and complications are difficult to translate. This is one of the limitations of PICO. Herskovic et al. stated that educators and PubMed user interface researchers should not focus on specific topics, but on overall efficient use of the system [15]. It is not feasible or practical to create versions of PICO adapted for all possible medical questions. As PICO is a method to break down a question into several concepts it might be useful to break down the question into several concepts regardless of the topic. We show that creating a PubMed query using four or five relevant terms is a good option to start with, regardless of the search topic. Using search tools may increase the search results further but we could not prove this because of the limited use of advanced search tools.

### Abstract selection in relation to query evaluation, retrieved articles and terms

The number of articles retrieved by a query showed a nearly logarithmic distribution, comparable to previous results[15]. The fact that rarely more than the first ten results were evaluated is an important finding. Previous research has shown that searchers seldom view more than 20 results when using search engines with relevance ranking[14]. Because such engines are likely to display the most relevant results on the first page, this can be a reasonable strategy. PubMed, however, does not perform relevance ranking, but by default displays the articles roughly by publication date in PubMed, beginning with the most recent. It is also possible to sort articles by author, actual publication date, journal and title but not according to relevance to the query. The chance of finding a relevant abstract within a list of several hundreds of article titles sorted by publication date, when only a fraction of the result is reviewed, is very low. Given the number of articles viewed on average by our population, the percentage of queries resulting in abstract selection started to decline rapidly with queries yielding more than 161 articles. The number of articles retrieved by a query is influenced by the number of terms used. Although using more relevant terms will usually result in a more accurate search result, using more terms increases the risk that the query will yield no results or no relevant results. The decline in the number of abstracts viewed when more than 5 terms are used can be explained by this phenomenon. The question is whether the fact that 4 or 5 terms in a query are optimal can be wholly attributed to the number of articles retrieved by a query. As both term count and number of articles retrieved affect the viewing of abstracts, one factor cannot be attributed entirely to the other.

### The query in relation to the search process

We investigated single queries, but the entire search process usually consists of sequential steps that should lead to an answer. After a PubMed query retrieves a set of articles the searcher may choose to evaluate a certain percentage of the abstracts and full-texts, but may also decide to refine the query. If the result is too large the query may be refined using hedges or more terms. If the result is too small the searcher may choose to remove terms that are too specific or expand terms with the "or" operator. The effects of these different measures are difficult to predict, especially if several options are combined. It is not surprising that using more relevant terms in a query will lead to fewer articles in the result, increasing the chance of article evaluation. The fact that four or five terms were optimal and fewer than 161 articles were optimal was an important finding. A previous study, describing the implementation of a Medline search tool for handhelds in a clinical setting, reported optimal values for term count and retrieved articles comparable to our results [26]. Knowing the optimal values can help in the design of search interfaces that promote the use of multiple terms in a query and the use of search tools, but can also aim for an optimal number of retrieved articles. Presenting the first ten unsorted results of several thousand articles is not useful for searching physicians. Analysis of queries that did not retrieve a sensible number of articles can help to guide the physician to increase the accuracy of the query, thus increasing the chance of retrieving a reasonable number of articles.

### Limitations

We observed Dutch physicians. As English is not their native language they may have used erroneous terms, which is likely to result in more queries with no articles in the result.

A possible source of error is that PubMed is our default database for searching. If a physician entered a query for UpToDate but forgot to select UpToDate as the search database, the query was sent to PubMed. Sending a query containing one term to other databases is usually sufficient, so the number of single term queries sent to PubMed might have been overestimated.

Our observation that the effect of using Mesh and limits is comparable to that of using adequate terms in a query is consistent with previous research [27,28].

We treated all queries as single entities and did not focus on the process of refining them. There is no way that a previous query can influence the articles retrieved by the next, so it cannot influence the next query result. Article selection might depend on experience from previous queries. Articles that were scanned in the first query will not be scanned in the second regardless of relevance to the question, so selection of articles in previous queries is not likely to result in bias.

Because we have observed natural behaviour by physicians in a very specific setting, our results are likely to be influenced by many factors and different ones may be obtained in different settings, limiting their generalizability.

## Conclusion

Our study is new in performing a detailed observation of the PubMed search process during busy medical practice in a hospital setting. Physicians at our hospital make very simple queries, containing fewer than three terms, and 31% result in viewing of abstracts. Search tools increased the selection of abstracts moderately to 39%. Both term use and number of retrieved articles influence abstract selection. Queries containing four or five terms yielding 2–161 articles were most effective in our population, with 63% abstract-viewing. PICO and other methods for improving query formulation should be the focus of more research and teaching, as this is likely to help considerably in improving search results during daily medical practice. Search engines aimed at on-the spot searching should analyze queries and give advice how to improve queries that retrieve too few or too many results instead of displaying the titles of the articles retrieved.

## Competing interests

The authors declare that they have no competing interests.

## Authors' contributions

AH, PV, AS and AO conceived and designed the study. The design of the internet portal was coordinated by AH and supervised by PV and AO. AH was responsible for data acquisition, interpretation and analysis. AH drafted the manuscript. All authors were involved in its critical revision and final approval for publication.

## Pre-publication history

The pre-publication history for this paper can be accessed here:


